# Sparsity-based method for ring artifact elimination in computed tomography

**DOI:** 10.1371/journal.pone.0268410

**Published:** 2022-06-28

**Authors:** Mona Selim, Essam A. Rashed, Mohammed A. Atiea, Hiroyuki Kudo

**Affiliations:** 1 Department of Computer Science, Faculty of Computers and Information, Suez University, Suez, Egypt; 2 Department of Mathematics, Faculty of Science, Suez Canal University, Ismailia, Egypt; 3 Graduate School of Information Science, University of Hyogo, Kobe, Japan; 4 Division of Information Engineering, Faculty of Engineering, Information and Systems, University of Tsukuba, Tsukuba, Japan; Tezpur University, INDIA

## Abstract

Ring artifact elimination is one of the popular problems in computed tomography (CT). It appears in the reconstructed image in the form of bright or dark patterns of concentric circles. In this paper, based on the compressed sensing theory, we propose a method for eliminating the ring artifact during the image reconstruction. The proposed method is based on representing the projection data by a sum of two components. The first component contains ideal correct values, while the latter contains imperfect error values causing the ring artifact. We propose to minimize some sparsity-induced norms corresponding to the imperfect error components to effectively eliminate the ring artifact. In particular, we investigate the effect of using different sparse models, *i.e.* different sparsity-induced norms, on the accuracy of the ring artifact correction. The proposed cost function is optimized using an iterative algorithm derived from the alternative direction method of multipliers. Moreover, we propose improved versions of the proposed algorithms by incorporating a smoothing penalty function into the cost function. We also introduce angular constrained forms of the proposed algorithms by considering a special case as follows. The imperfect error values are constant over all the projection angles, as in the case where the source of ring artifact is the non-uniform sensitivity of the detector. Real data and simulation studies were performed to evaluate the proposed algorithms. Results demonstrate that the proposed algorithms with incorporating smoothing penalty and their angular constrained forms are effective in ring artifact elimination.

## 1 Introduction

**Fig 1:**
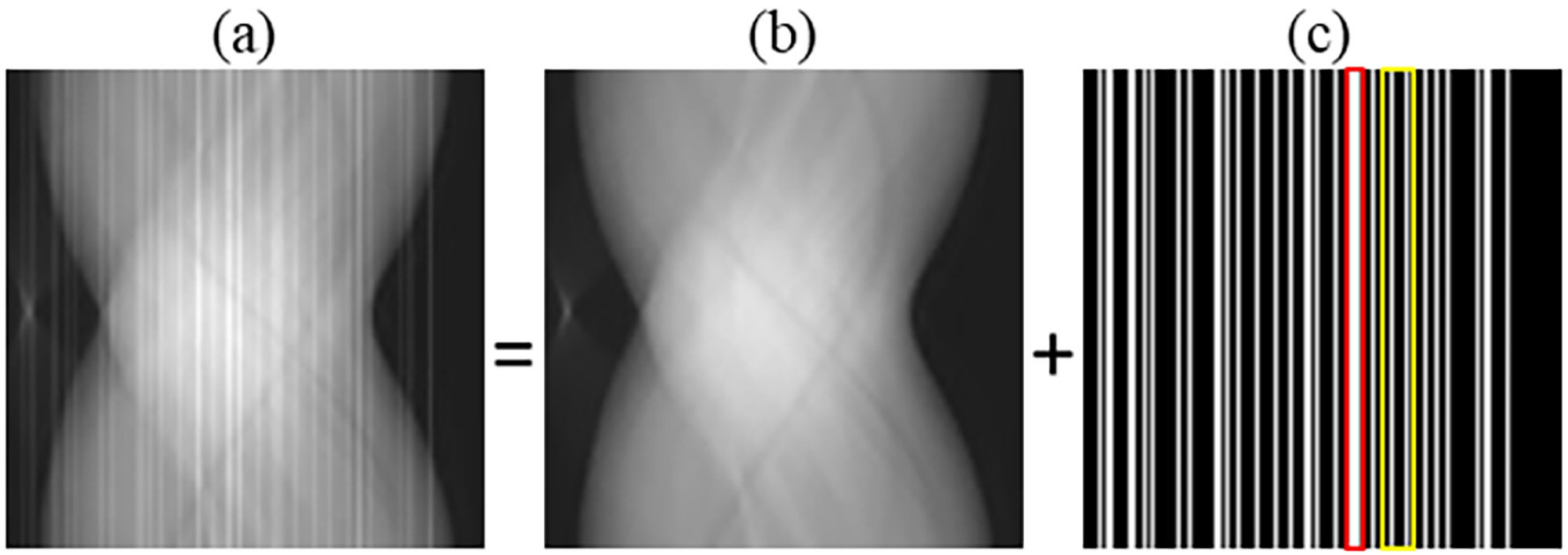
Projection data with vertical artifacts caused by bin failures in (a) can be modeled as a combination of (b) ideal projection data and (c) ring artifact in the projection data domain. Red and yellow rectangles in (c) demonstrate the adjacent and non-adjacent bins failure cases, respectively.

Ring artifact is one of the well-known image degradations that restrict the quality of X-ray computed tomography (CT) images [1]. In addition to general-purpose medical CT scanners, it also occurs in CT images that are generated from a flat-panel detector such as C-arm CT, dental CT, and micro-CT. The ring artifact can be recognized in the image domain as bright or dark patterns of concentric circles. These circles vary in intensity value from ring to ring. Also, it can be interpreted in the sinogram domain as bright or dark vertical lines having different intensity values. The ring artifact mainly arises as a consequence of some physical factors associated with non-uniform detector sensitivity, such as 1) existence of imperfect or dead detector elements, 2) incomplete calibration of the detector elements, 3) impurity or dust in the scintillator screen, or 4) presence of variation in the hardness or intensity of x-ray beams. The ring artifact may resemble some structure of the scanned object, which prevents the resulting image from being used in various applications. Hence, the elimination of ring artifact is a significant issue. One conventional technique that is used in ring artifact elimination is called flat-field correction. It was reported that such simple technique could eliminate ring artifact partially [2]. Other methods that deal with the ring artifact problem can be divided into hardware- and software-based methods. The removal of ring artifact in most software-based methods was performed before or after the reconstruction process. However, such methods may introduce other artifacts. On the other hand, the elimination of the ring artifact during the reconstruction overcomes the limitations of such methods. In this paper, inspired by the compressed sensing (CS) theory [3], we propose a software-based method to estimate and eliminate the ring artifact during the image reconstruction process. Some sparsity-based methods were presented to suppress the ring artifacts [4–8]. In this work, we introduce using sparsity-induced norms like *ℓ*_0_, Huber *ℓ*_0_, and Huber *ℓ*_1_ in ring artifact removal through the reconstruction steps. The sinogram data corresponding to the image containing the ring artifact is also contaminated by incorrect measurement values similar to the example shown in Fig 1. The sinogram data in such case is composed of the perfect ideal values and the imperfect error values causing the ring artifact. The contribution of this paper is as follows. First, we propose to use some sparsity-induced norms of the imperfect component as data fidelity to enforce the sparsity of the imperfect component to eliminate the ring artifact. Second, we investigate the effect of using different sparsity-induced norms on the ring artifact elimination. Third, we propose to improve image quality in the proposed algorithms by incorporating a smoothing penalty function into the cost function. Fourth, we study a particular case of the developed algorithms, where the values of the imperfect error component are constant over all the projection angles. Finally, we remark that this work can be considered a particular application of the use of sparsity-induced norms in data fidelity to eliminate the effect of abnormal error data in the sinogram [9, 10]. Such approach was proposed in our previous work together with a special application to the metal artifact reduction problem [11]. This paper demonstrates that the similar approach works well for the ring artifact correction problem.

This paper is organized as follows. The related work is presented in Section 2. The proposed algorithms are detailed in Section 3. After that, we show the results of the experimental studies in Section 4. Finally, the conclusion is provided in 5.

## 2 Related work

There are numerous methods that have been investigated to suppress the ring artifact. These methods can be classified into two general categories. The first category contains methods based on the modification of hardware, such as those detailed in [12, 13]. The second category contains software-based methods. Due to the necessity of modifying the device hardware and consequently the extra cost in the first class, most of the investigated methods belong to the second category. The software-based methods can be further divided into three classes as follows:

- Pre-processing methods: remove the vertical stripes from the sinogram before image reconstruction.
- Post-processing methods: remove the ring artifact from the image after image reconstruction.
- In-processing methods: remove the ring artifact during image reconstruction.

### 2.1 Pre-processing methods

A technique based on a combination of wavelet decomposition and Fourier filtering was investigated to eliminate the stripes from the sinogram [14]. Also, a sinogram-based method was proposed by supposing smoothness of the detector measurements for every projection angle [15]. A derivative-based algorithm was developed to detect the mis-calibrated and defective detector elements [16]. This algorithm used a bias correction model and a weighted moving average filter to remove the stripe artifacts. An approach focused on filtering scheme by morphological operations was developed for the ring artifact elimination [17]. A detector line ratio method based on computing the ratio between the neighboring detector elements was proposed in the sinogram space [18]. Furthermore, based on classifying the stripe artifacts in the sinogram into different types, three methods were designed to deal with each type individually [19]. Also, a combination of these three methods was proposed, and they concluded that this combination could suppress all types of artifacts. A ring artifact removal algorithm was proposed in the form of a 1D filter and can be combined with the standard filter used in the FBP method [20]. Using wavelet filtering and weighted moving average filter in the sinogram space, an approach was presented to remove the ring artifact [21]. The removal of stripe artifacts in the sinogram was performed via variable window averaging and weighted moving average filters [22]. A U-Net-like deep neural network was employed to reduce the stripe artifacts in the sinogram space [23]. However, filtering the sinogram to remove the vertical stripes may introduce new artifacts.

### 2.2 Post-processing methods

A correction algorithm was proposed by utilizing mean and median filters in both Cartesian and polar coordinate systems. They concluded that implementing their algorithm in the polar coordinate system gives higher performance [24]. A method based on generative adversarial networks was investigated to remove the ring artifact from the image in the Cartesian coordinates [25]. Moreover, several methods have been investigated to suppress the ring artifact from the image in the polar coordinate system. For example, an iterative method was developed to remove the ring artifact from CT images utilizing relative total variation (TV) [26]. A method using independent component analysis was presented for suppressing the ring artifact [27]. This method divides the image into many independent components. Then, the components that include the rings are identified to apply a smoothing filter only on the ring components. A polar wavelet Gaussian filtering technique was presented to suppress the ring artifact [28]. Furthermore, a method that depends on TV-Stokes and unidirectional TV model was introduced to eliminate the ring artifact from cone-beam CT images [29]. Based on a unidirectional relative variation model, an approach was investigated to suppress the ring artifact [30]. Also, a variation-based method was formulated using *ℓ*_0_-norm/*ℓ*_1_-norm regularization term to remove the ring artifact from the reconstructed images [4]. The reduction of ring artifact was achieved based on sparse domain regularized stripe decomposition technique and guided image filtering [5]. Moreover, a radial basis function neural network was designed to eliminate the ring artifact from the reconstructed images [6]. Using generative adversarial networks and unidirectional relative TV, a method was presented to suppress the artifact from the CT images [31]. Most of the post-processing methods consist of the following steps. Transform the image contaminated with the ring artifact to the polar coordinates and carry out the filtering, then return to the original Cartesian coordinates. The rationale behind using such transformation is as follows. The ring artifact in the Cartesian coordinates can be converted into lines in the polar coordinates, and filtering out the straight lines is much easier than the rings. However, image quality achieved by these methods relies on the accurate determination of the center of the ring artifact. This is because a small difference in the center determination could convert the rings into curvy lines instead of the straight lines. Such curvy lines distort the output image in the Cartesian coordinates.

### 2.3 In-processing methods

An optimization method based on TV and dictionary learning was investigated to correct the ring artifact [7]. Recently, an algorithm for the ring artifact reduction was proposed using ring TV and correction coefficient [8]. The proposed method follows this class of methods, where removing the ring artifact during the reconstruction process does not require any data modification before or after the reconstruction. Also, the probability of introducing other distortions like the pre-or post-processing methods is low.

## 3 Methods

### 3.1 Problem formulation

In the case of ring artifact existence in the image, the image reconstruction model in x-ray CT can be described as follows:

$$\begin{array}{c} A\vec{x}=\vec{b}+\vec{\lambda }, \end{array}$$ (1)

where $\vec{b}={\left(b_{1},b_{2},\ldots ,b_{M}\right)}^{⊤}$ is the projection data. Also, $\vec{\lambda }={\left(\lambda_{1},\lambda_{2},\ldots ,\lambda_{M}\right)}^{⊤}$ is the imperfect error component of projection data, which may have positive or negative values. The size of both $\vec{b}$ and $\vec{\lambda }$ is *M*, where *M* = *R* × Θ (*R* represents the number of detector bins, and Θ represents the number of angles). Also, $\vec{x}={\left(x_{1},x_{2},\ldots ,x_{N}\right)}^{⊤}$ is a vector representing a 2D image consisting of *N* (pixels), and *A* = {*a*_*ij*_} denotes the *M* × *N* system matrix. From the physical model of ring artifact generation in CT, it is a reasonable assumption that the values of most elements in $\vec{\lambda }$ are zeros, and only a small number of non-zero imperfect elements exist in $\vec{\lambda }$. Consequently, the imperfect vector $\vec{\lambda }$ can be considered as a sparse vector. The sparsity of imperfect vector $\vec{\lambda }$ inspires us to use the CS theory in ring artifact elimination problem. We exploit the CS theory in our work in order to estimate the imperfect data that cause the ring artifact and eliminate them from the projection data during image reconstruction to obtain a ring-free image. Therefore, we propose to formulate a cost function in image reconstruction by utilizing some sparsity-induced norms of $\vec{\lambda }$ as data fidelity to enforce the sparsity of imperfect component as follows:

$$\begin{array}{c} {\text{Minimize}}_{\left(\vec{x},\vec{\lambda }\right)}\;\;\;S\left(\vec{\lambda }\right)\;\;\;\text{subject}\;\text{to}\;\;\;A\vec{x}-\vec{b}=\vec{\lambda }, \end{array}$$ (2)

where $S(\vec{\lambda })$ can be one of well-known sparse models in CS such as *ℓ*_1_-norm or *ℓ*_0_-norm. The vector $\vec{\lambda }$ in Eq (2) is described to include only the non-ideal elements that generate the ring artifact. However, in real CT imaging, both the statistical noise and imperfect error values are included together in $\vec{\lambda }$. In such case, Huber loss function can be utilized instead of *ℓ*_1_-norm as a sparse model because it is a hybrid of *ℓ*_1_-norm and *ℓ*_2_-norm. Throughout the paper, we call it Huber *ℓ*_1_-norm. By the same manner, we suggest defining Huber *ℓ*_0_-norm as a combination of *ℓ*_0_-norm and *ℓ*_2_-norm. In summary, $S(\vec{\lambda })$ can be one of the following functions:

$$\begin{array}{c} S\left(\vec{\lambda }\right)={\left\|\vec{\lambda }\right\|}_{1}^{1}\;\;\;\;\;\;\;\;\;\;\;\;\;\;\left(\ell_{1}\text{-norm}\right), \end{array}$$ (3)

$$\begin{array}{c} S\left(\vec{\lambda }\right)={\left\|\vec{\lambda }\right\|}_{0}^{0}\;\;\;\;\;\;\;\;\;\;\;\;\;\;\left(\ell_{0}\text{-norm}\right), \end{array}$$ (4)

$$\begin{array}{ccc} S(\vec{\lambda }) & = & \sum_{i=1}^{M}s(\lambda_{i}),\;\;\;\;s\left(\lambda \right)=\{\begin{array}{cc} \lambda^{2}/2 & |\lambda |<\delta \\ \delta |\lambda |+\delta^{2}/2 & |\lambda |\geq \delta \end{array}\;\;\left(\text{Huber}\;\ell_{1}\text{-norm}\right), \end{array}$$ (5)

$$\begin{array}{ccc} S(\vec{\lambda }) & = & \sum_{i=1}^{M}s(\lambda_{i}),\;\;\;\;s\left(\lambda \right)=\{\begin{array}{cc} \lambda^{2}/2 & |\lambda |<\delta \\ {\delta |\lambda |}_{0}+\delta^{2}/2 & |\lambda |\geq \delta \end{array}\;\;\left(\text{Huber}\;\ell_{0}\text{-norm}\right). \end{array}$$ (6)

Generally, it is clear from the different causes of ring artifact that the values of the imperfect vector in the projection data may be dependent on the measured angles. In other words, the imperfect values are variables for all the angles. In addition to this general case, we suggest studying a special case where the ring artifact originates from physical factors related to hardware, such as the existence of some imperfect detector bins. Then, the imperfect elements in $\vec{\lambda }$ occur due to the imperfect detector bins. In CT, if some detector bins have errors, the incorrect values corresponding to those bins will be repeated for all the angles *θ*. Consequently, the measured value of the imperfect vector $\vec{\lambda }$ at any detector bin is constant for all the angles *θ*. Hereafter, such model having constant imperfect elements in the angular direction is called angular constrained form. It can be mathematically expressed as follows. Let us express the system matrix *A* using submatrices corresponding to each sinogram angle as *A* = [*A*_1_, *A*_2_, …, *A*_Θ_], where *A*_*i*_ denotes the system matrix corresponding to the *i*-th projection angle. Similarly, we express the projection data as $\vec{b}={\left({\vec{b}}_{1},{\vec{b}}_{2},\ldots ,{\vec{b}}_{\Theta }\right)}^{T}$, where ${\vec{b}}_{i}$ denotes the projection data corresponding to the *i*-th projection angle. Using these notations, the image reconstruction problem in the angular constrained form can be formulated as

$$\begin{array}{c} \text{Minimize}\;\;\;S\left(\vec{\lambda }\right)\;\;\;\text{subject}\;\text{to}\;\;\;A_{\theta }\vec{x}-\vec{b_{\theta }}=\vec{\lambda },\forall \theta . \end{array}$$ (7)

We remark that $\vec{\lambda }$ is constant for all *θ*, which originates from the constraint that values of elements in the imperfect vector are same for all the angles.

### 3.2 Proposed algorithms

The formulated cost function can be optimized using any iterative algorithm. For example, first-order primal-dual method, alternative direction method of multipliers (ADMM) [32], and proximal splitting. In this work, we use ADMM method as a prototype example to develop iterative algorithms to solve the formulated cost function. We start with using *ℓ*_1_-norm as an example of the sparsity-induced norm $S(\vec{\lambda })$ in the proposed cost function as follows:

$$\begin{array}{c} {\text{Minimize}}_{\left(\vec{x},\vec{\lambda }\right)}\;\;\;{\left\|\vec{\lambda }\right\|}_{1}^{1}\;\;\;\text{subject}\;\text{to}\;\;\;A\vec{x}-\vec{b}=\vec{\lambda } \end{array}$$ (8)

The augmented Lagrangian corresponding to Eq (8) is

$$\begin{array}{c} L_{\rho }\left(\vec{x},\vec{\lambda },\vec{u}\right)=\;\|\vec{\lambda }{\|}_{1}^{1}\;+\frac{\rho }{2}{\left\|\vec{\lambda }-A\vec{x}+\vec{b}+\vec{u}\right\|}_{2}^{2}, \end{array}$$ (9)

where $\vec{u}$ denotes the Lagrange multiplier and *ρ* > 0 is a positive constant. Using the augmented Lagrangian function in Eq (9), ADMM method finds the solution to the primal-dual problem $\left(\vec{x},\vec{u}\right)$ according to the following three steps:

$$\begin{array}{c} {\vec{x}}^{k+1}=\text{arg}\;{\text{min}}_{\vec{x}}\;\;\frac{\rho }{2}{\left\|{\vec{\lambda }}^{k}-A{\vec{x}}^{k}+\vec{b}+{\vec{u}}^{k}\right\|}_{2}^{2}, \end{array}$$ (10)

$$\begin{array}{c} {\vec{\lambda }}^{k+1}=\text{arg}\;{\text{min}}_{\vec{\lambda }}\;\;\|\vec{\lambda^{k}}{\|}_{1}^{1}+\frac{\rho }{2}{\left\|{\vec{\lambda }}^{k}-A{\vec{x}}^{k+1}+\vec{b}+{\vec{u}}^{k}\right\|}_{2}^{2}, \end{array}$$ (11)

$$\begin{array}{c} {\vec{u}}^{k+1}={\vec{u}}^{k}+\left({\vec{\lambda }}^{k+1}-A{\vec{x}}^{k+1}+\vec{b}\right). \end{array}$$ (12)

The update of image $\vec{x}$ is performed through the minimization in Eq (10). Since $D\left(\vec{x}\right)=\frac{\rho }{2}{\left\|{\vec{\lambda }}^{k}-A{\vec{x}}^{k+1}+\vec{b}+{\vec{u}}^{k}\right\|}_{2}^{2}$ is a differentiable function in this case, it can be minimized using the standard gradient descent (GD) method as follows:

$$\begin{array}{c} {\vec{x}}^{l+1}={\vec{x}}^{l}-\alpha \nabla_{\vec{x}}D\left({\vec{x}}^{l}\right),\;\;\;l=1,2,\ldots ,L, \end{array}$$ (13)

where *α* > 0 denotes the step size, *L* is the maximum number of iterations in the GD method, and $\nabla_{x}D\left({\vec{x}}^{l}\right)$ is calculated as follows:

$$\begin{array}{c} \nabla_{x}D\left({\vec{x}}^{l}\right)=-\rho A^{T}\left(\left(\vec{\lambda }+\vec{b}+\vec{u}\right)-A{\vec{x}}^{l}\right). \end{array}$$ (14)

With respect to the update of the imperfect error vector $\vec{\lambda }$, it can be done by the minimization in Eq (11). By recalling the definition of proximity operator as follows:

$$\begin{array}{c} {\text{Prox}}_{\mu f}\left(\vec{y}\right)=\text{arg}\;{\text{min}}_{\vec{x}}\;\mu f\left(\vec{x}\right)+\frac{1}{2}{\left\|\vec{x}-\vec{y}\right\|}_{2}^{2}, \end{array}$$ (15)

the necessary computation in Eq (11) is reduced to

$$\begin{array}{c} {\text{Prox}}_{\mu \|\vec{\lambda^{k}}{\|}_{1}^{1}}\left({\vec{d}}^{k}\right), \end{array}$$

$$\begin{array}{c} {\vec{d}}^{k}=A{\vec{x}}^{k+1}-\vec{b}-{\vec{u}}^{k} \end{array}$$

and *μ* = 1/*ρ*. It can be solved directly using a simple formula in closed form via the soft thresholding [33] function as follows:

$$\begin{array}{c} {\vec{\lambda }}^{k+1}=\text{soft-thresholding}\left({\vec{d}}^{k},\mu \right), \end{array}$$ (16)

where the soft-thresholding(⋅) is defined by

$$\begin{array}{c} \;\;\;\text{soft-thresholding}\left(a,b\right)=\{\begin{array}{cc} a-b & a>b\\ a+b & a<-b\\ 0 & otherwise \end{array}. \end{array}$$ (17)

The final iterative algorithm can be summarized in Algorithm 1.

**Algorithm 1** *ℓ*_1_ **-ring algorithm.**

Input the projection data $\vec{b}$.

Set initial value for ${\vec{x}}^{1}$, ${\vec{\lambda }}^{1}$ and ${\vec{u}}^{1}$. Set the parameters (*α* > 0 and *ρ* > 0).

**for** *k* = 1, 2, …, *K* **do**

${\vec{y}}^{1}={\vec{x}}^{k}$

 **for** *l* = 1, 2, …, *L* **do**

${\vec{y}}^{l+1}={\vec{y}}^{l}-\alpha \nabla_{\vec{y}}D\left({\vec{y}}^{l}\right)$

 **end**

${\vec{x}}^{k+1}={\vec{y}}^{L}$

${\vec{\lambda }}^{k+1}=Prox_{\left(1/\rho \right)\|\vec{\lambda^{k}}{\|}_{1}^{1}}\left(A{\vec{x}}^{k+1}-\vec{b}-{\vec{u}}^{k}\right)$

${\vec{u}}^{k+1}={\vec{u}}^{k}+{\vec{\lambda }}^{k+1}-A{\vec{x}}^{k+1}+\vec{b}$

**end**

Output the reconstructed image $\vec{x}$.

We followed the same steps to derive three iterative algorithms corresponding to the remaining three sparsity-induced norms mentioned above, *i.e.* *ℓ*_0_-norm, Huber *ℓ*_1_-norm and Huber *ℓ*_0_-norm. The result of the derivation shows the following. Almost same algorithms can be obtained for the cases of using four different norms, and the only difference among the four cases appears in the form of the proximity operator in Step 2. For clarity, the proximity operators corresponding to the four cases are summarized in Table 1. Throughout the paper, we call the algorithms corresponding to *ℓ*_1_-norm, *ℓ*_0_-norm, Huber *ℓ*_1_-norm, and Huber *ℓ*_0_-norm by *ℓ*_1_-ring, *ℓ*_0_-ring, H*ℓ*_1_-ring, and H*ℓ*_0_-ring algorithms, respectively.

**Table 1:** Proximity operators corresponding to different sparsity-induced norms.

*f*(*x*)	Prox_*μf*_ (*y*)
*ℓ*_1_-norm	$\{\begin{array}{cc} y-\mu & y>\mu \\ y+\mu & y<-\mu \\ 0 & Otherwise \end{array}$
*ℓ*_0_-norm	$\{\begin{array}{cc} t & y^{2}/2>\mu \\ 0 & Otherwise \end{array}$
Huber *ℓ*_1_-norm	$\{\begin{array}{cc} y+\mu \delta & y<-(\mu +1)\delta \\ y/(\mu +1) & -(\mu +1)\delta \leq y\leq (\mu +1)\delta \\ y-\mu \delta & y>(\mu +1)\delta \end{array}$
Huber *ℓ*_0_-norm	$\{\begin{array}{cc} y/(\mu +1) & -(\mu +1)\delta \leq y\leq (\mu +1)\delta \\ y & Otherwise \end{array}$

### 3.3 Extension with smoothing penalty

We propose to incorporate a smoothing penalty $H(\vec{x})$ into the proposed cost function in Eq (2) to strengthen the power to remove the artifact. The new cost function with the smoothing penalty is given by

$$\begin{array}{c} {\text{Minimize}}_{\left(\vec{x},\vec{\lambda }\right)}\;\;\;\;S\left(\vec{\lambda }\right)+\beta H\left(\vec{x}\right)\;\text{subject}\;\text{to}\;A\vec{x}-\vec{b}=\vec{\lambda }, \end{array}$$ (18)

where *β* > 0 denotes the hyper-parameter that controls the strength of smoothing. In this work, we choose to use a smoothing penalty similar to Markov random fields (MRFs) [34]. This is because it possesses the advantage of edge-preserving property, and it is also a differentiable function that can be easily minimized. With respect to the optimization of penalized cost function, we first describe the *ℓ*_1_-norm case, followed by explaining how it can be changed to the other three norms. The cost function in the *ℓ*_1_-norm case is given by

$$\begin{array}{c} {\text{Minimize}}_{\left(\vec{x},\vec{\lambda }\right)}\;\;\;\;{\left\|\vec{\lambda }\right\|}_{1}^{1}+\beta H\left(\vec{x}\right)\;\text{subject}\;\text{to}\;A\vec{x}-\vec{b}=\vec{\lambda }, \end{array}$$ (19)

where $H(\vec{x})$ is the smoothing penalty defined by

$$\begin{array}{c} H\left(\vec{x}\right)=\sum_{j}\sum_{\tilde{j}\in U_{j}}w_{j\tilde{j}}h\left(x_{j}-x_{\tilde{j}}\right), \end{array}$$ (20)

$$\begin{array}{c} h\left(t\right)=\{\begin{array}{cc} t^{2}/2 & |t|<\eta \\ \eta |t|+\eta^{2}/2 & |t|\geq \eta \end{array}, \end{array}$$ (21)

where *U*_*j*_ denotes the set of eight neighboring pixels around the *j*-th pixel, and $w_{j\tilde{j}}$ is the weighting parameter computed as the Euclidean distance between the *j*-th pixel and the $\tilde{j}$-th pixel. This modified cost function can be optimized by using iterative algorithms derived from ADMM similar to those in the previous section. The same ADMM steps yield, except Step 1, which can be modified and minimized as follows:

$$\begin{array}{c} {\vec{x}}^{l+1}={\vec{x}}^{l}-\alpha \nabla_{\vec{x}}D\left({\vec{x}}^{l}\right)-\beta^{\prime }\nabla_{\vec{x}}H\left({\vec{x}}^{l}\right),\;\;\;l=1,2,\ldots ,L, \end{array}$$ (22)

where $\beta^{\prime }=\alpha \beta$, and $\nabla_{\vec{x}}H\left({\vec{x}}^{l}\right)$ is calculated as follows:

$$\begin{array}{c} \nabla_{\vec{x}}H\left(\vec{x}\right)=\sum_{j}\sum_{\tilde{j}\in U_{j}}w_{j\tilde{j}}\{\begin{array}{cc} x_{j}-x_{\tilde{j}} & |x_{j}-x_{\tilde{j}}|<\eta \\ \eta & x_{j}-x_{\tilde{j}}\geq \eta \\ -\eta & x_{j}-x_{\tilde{j}}\leq -\eta \end{array} \end{array}$$ (23)

The final algorithm is detailed in Algorithm 2.

**Algorithm 2** *ℓ*_1_ **-smth-ring algorithm.**

Input the projection data $\vec{b}$.

Set initial value for ${\vec{x}}^{1}$, ${\vec{\lambda }}^{1}$ and ${\vec{u}}^{1}$. Set the parameters (*α* > 0, *ρ* > 0 and $\beta^{\prime }$).

**for** *k* = 1, 2, …, *K* **do**

${\vec{y}}^{1}={\vec{x}}^{k}$

 **for** *l* = 1, 2, …, *L* **do**

${\vec{y}}^{l+1}={\vec{y}}^{l}-\alpha \nabla_{\vec{y}}D\left({\vec{y}}^{l}\right)-\beta^{{}^{\prime }}\nabla_{\vec{y}}H\left({\vec{y}}^{l}\right)$

 **end**

${\vec{x}}^{k+1}={\vec{y}}^{L}$

${\vec{\lambda }}^{k+1}={\text{Prox}}_{\left(1/\rho \right)\|\vec{\lambda^{k}}{\|}_{1}^{1}}\left(A{\vec{x}}^{k+1}-\vec{b}-{\vec{u}}^{k}\right)$

${\vec{u}}^{k+1}={\vec{u}}^{k}+{\vec{\lambda }}^{k+1}-A{\vec{x}}^{k+1}+\vec{b}$

**end**

Output the reconstructed image $\vec{x}$.

Similarly to the case without the smoothing penalty, the iterative algorithms corresponding to the other three norms mentioned above are given as follows: change the proximity operator in Algorithm 2, as shown in Table 1. Throughout the paper, we call the four obtained algorithms by *ℓ*_1_-smth-ring, *ℓ*_0_-smth-ring, H*ℓ*_1_-smth-ring, and H*ℓ*_0_-smth-ring algorithms, respectively. In addition, we call a collection of these four algorithms using the smoothing penalty by improved algorithms to distinguish them from the algorithms without the smoothing.

### 3.4 Constrained algorithms

This section describes iterative algorithms for the angular constrained form where elements of imperfect error vector have the same values for all the angles, *i.e.* the formulation of Eq (7). A simple algorithm for this special case can be given as follows. The proposed algorithms in the previous sections are only modified by including the constraint that the values of elements in the imperfect vector $\vec{\lambda }$ are constant for all the angles (the angular-independency constraint). We can observe the following with a deep look at Step 4 of the iterative algorithms at an iteration *k*. Each element λ_*rθ*_ of the imperfect vector $\vec{\lambda }$ are updated for every detector bin (*r*) in every projection angle (*θ*) based on each elements *d*_*rθ*_ of the vector $\vec{d}$ defined above as follows:

for *r* = 1, 2, …, *R* do

 for *θ* = 1, 2, …, Θ do

  $\lambda_{r\theta }^{k+1}={\text{Prox}}_{\mu \|\vec{\lambda^{k}}{\|}_{1}^{1}}\left(d_{r\theta }^{k}\right)$

 end

end

Applying the angular-independency constraint means that the update of $\vec{\lambda }$ occurs only at every detector bin as follows:

for *r* = 1, 2, …, *R* do

 $\lambda_{r}^{k+1}={\text{Prox}}_{\mu \|\vec{\lambda^{k}}{\|}_{1}^{1}}\left(\left(\sum_{\theta =1}^{\Theta }d_{r\theta }^{k}\right)/\Theta \right)$

 for *θ* = 1, 2, …, Θ do

  $\lambda_{r\theta }^{k+1}=\lambda_{r}^{k+1}$

 end

end

Using the update of $\vec{\lambda }$ mentioned above in each iterative algorithm described in the previous sections, the corresponding iterative algorithm including the angular-independency can be obtained. We call the resulting algorithms by (cstr-*ℓ*_1_-ring, cstr-*ℓ*_0_-ring, cstr-H*ℓ*_1_-ring, and cstr-H*ℓ*_0_-ring algorithms) for the case without the smoothing, and (cstr-*ℓ*_1_-smth-ring, cstr-*ℓ*_0_-smth-ring, cstr-H*ℓ*_1_-smth-ring, and cstr-H*ℓ*_0_-smth-ring algorithms) for the case with the smoothing.

### 3.5 Convergence properties

The convergence of iterative algorithm using *ℓ*_1_-norm or Huber *ℓ*_1_-norm is guaranteed for the following reason. These algorithms can be considered as an application of ADMM to the convex minimization subject to the linear constraint. However, the iterative algorithms using *ℓ*_0_-norm or Huber *ℓ*_0_-norm are not guaranteed to converge due to the non-convex nature of cost functions. However, as we will show in the next section, the algorithms using *ℓ*_0_-norm or Huber *ℓ*_0_-norm converge to nice approximate solutions in practice. Also, their power to eliminate the ring artifact is much stronger compared to the *ℓ*_1_-norm based algorithms. This observation motivated us to keep the *ℓ*_0_-norm based algorithms in this paper in spite of the lack of mathematical guarantee in the convergence. We note that similar behaviors, *i.e.* approximate solution with the *ℓ*_0_-norm works better than the exact solution with the *ℓ*_1_-norm, have been observed in other application areas of CS.

## 4 Results

### 4.1 Experimental setup

To evaluate the proposed algorithms, we performed experimental studies using real data in addition to simulation data. In the real data, some experiments for propagation-based phase-contrast imaging were performed by synchrotron radiation X-ray micro-CT (Spring-8, BL20XU beamline), Japan [35, 36]. A sample of duplex stainless steel was scanned with an x-ray energy of 37.7 (KeV). The sample-to-detector distance was varied between 8 and 1200 (mm). Also, a CMOS camera of 4.0 megapixels, with a 10 (*μ*m) thick scintillator was used for measuring the projection data. The number of projection data was 1800 over 180 degrees, and each projection data consisted of 1024 × 1024 pixels. From the conventional FBP reconstruction of this data, the central slice of the reconstructed 3D image was contaminated with strong ring artifact. Moreover, in the simulation data, we used a 2D slice obtained from a dataset of screening chest CT images. The dataset includes 68 volumes for various patients scanned using Hitachi CT-W950SR scanner. Each volume is composed of 25 to 31 transaxial slices, where each slice is composed of 320 × 320 (pixels) with a slice thickness of 10 (mm) and a pixel size of 1 × 1 (mm). The 2D chest slice was resized to 512 × 512 (pixels). The simulation studies were carried out with and without adding noise to the projection data. It is known that the statistical noise in the CT measurements follows Poisson distribution [37]. Assume that *b*_0_ is the number of average photon counts transmitted from the x-ray source, and *b*_*i*_ is the detected photons measured after attenuation by the scanned object at detector bin *i*. Then, the noisy projection data *g*_*i*_ at detector bin *i* can be calculated using Eqs (24) and (25). Moreover, to simulate the ring artifact, we first converted the image into the sinogram by the forward projection. Then, the generated sinogram was altered by assuming that some selected detector bins have non-uniform sensitivity compared to normal bins. Finally, using the altered sinogram, we reconstructed the image by the standard filtered back-projection (FBP) method to obtain the corrupted image. For the purpose of performance evaluation, we compared our algorithms with the correction method [28] and the combination method [19]. We refer to these methods as M1 and M2, respectively. Three parameters are required to implement the M1 method (Gaussian filter width *σ*, decomposition level *L*, and wavelet base function *W*). On the other hand, to correct the image by the M2 method, some parameters need to be adjusted, such as the ratio between the defective and background values *R*. Also, the size of the smoothing filters that remove the small to medium and large stripes in the sinogram (*S*_*m*_, *S*_*l*_) is required for executing the M2 method. In order to obtain satisfactory results with the M1 and M2 methods, we selected their parameters based on the best image quality in terms of RRMSE values besides visual evaluation. Furthermore, to study the effect of using the standard *ℓ*_2_-norm on ring artifact removal, we compared our proposed algorithms with two algorithms using *ℓ*_2_-norm for the data fidelity term with and without smoothing penalty. Additionally, the GD method was used for the optimization problem of these two algorithms, which we name *ℓ*_2_ and *ℓ*_2_-smth algorithms. All the algorithms were implemented using C++ platform on a machine with Intel(R) core(TM) i7–2670QM CPU @ 2.20 GHz processor and 6 GB RAM. The average execution time of the proposed algorithms for every iteration was 6.0 seconds.

$$\begin{array}{c} g_{i}=-\text{log}\left(\frac{\tilde{b_{i}}}{b_{0}}\right), \end{array}$$ (24)

$$\begin{array}{c} \tilde{b_{i}}\approx \text{Poisson}\left(b_{i}=b_{0}{\text{exp}}^{-A_{i}\vec{x}}\right). \end{array}$$ (25)

### 4.2 Image quality measurements

To evaluate image quality achieved by the proposed algorithms, we used two image quality metrics throughout the simulation studies. Suppose that $\vec{z}={\left(z_{1},\ldots ,z_{N}\right)}^{⊤}$ denotes the reference image and $\vec{x}$ denotes the reconstructed or corrected image. The first metric is the relative root-mean-square error (RRMSE), which can be computed by

$$\begin{array}{c} \text{RRMSE}=\sqrt{\frac{\sum_{i=1}^{N}{\left(x_{i}-z_{i}\right)}^{2}}{\sum_{i}x_{i}}}. \end{array}$$ (26)

The second metric is the structural similarity (SSIM) index [38], which can be computed by

$$\begin{array}{c} \text{SSIM}=\frac{\left(2\mu_{x}\mu_{z}+c_{1}\right)\left(2\sigma_{xz}+c_{2}\right)}{\left(\mu_{x}^{2}+\mu_{z}^{2}+c_{1}\right)\left(\sigma_{x}^{2}+\sigma_{z}^{2}+c_{2}\right)}, \end{array}$$ (27)

where *μ*_*x*_ and *μ*_*z*_ are the mean values of images $\vec{x}$ and $\vec{z}$, respectively. $\sigma_{x}^{2}$ and $\sigma_{z}^{2}$ are the variance values of images $\vec{x}$ and $\vec{z}$, respectively. *σ*_*xz*_ is the correlation coefficient value of image $\vec{x}$ and image $\vec{z}$. *c*_1_ = (*k*1*s)^2^ and *c*_2_ = (*k*2*s)^2^ where *k*1 = 0.01 and *k*2 = 0.03 and *s* is the dynamic range of the pixel values.

### 4.3 Results of the simulation with noise-free projection data

**Fig 2:**
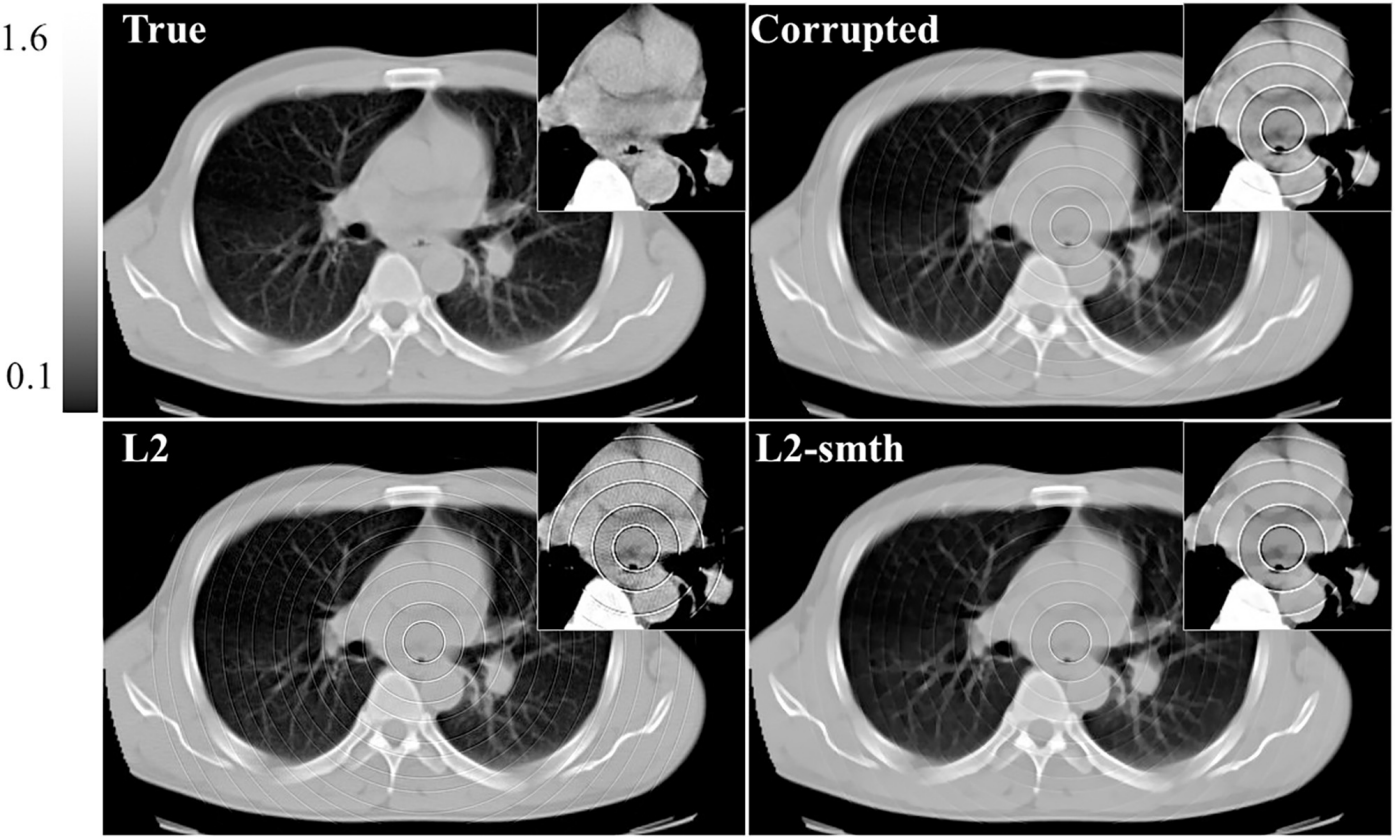
Reconstructed images using *ℓ*_2_ and *ℓ*_2_-smth algorithms for the first study case. All reconstructed images in figures hereafter are displayed with the same grayscale for consistency.

**Fig 3:**
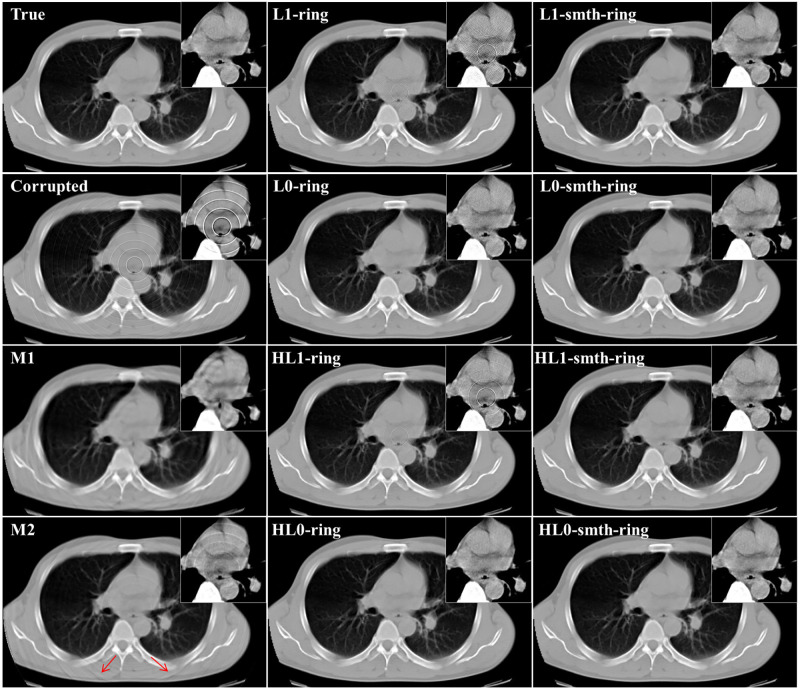
Reconstructed images in the first study case (noise-free, non-adjacent error detector bins, and angular-independent error case).

**Fig 4:**
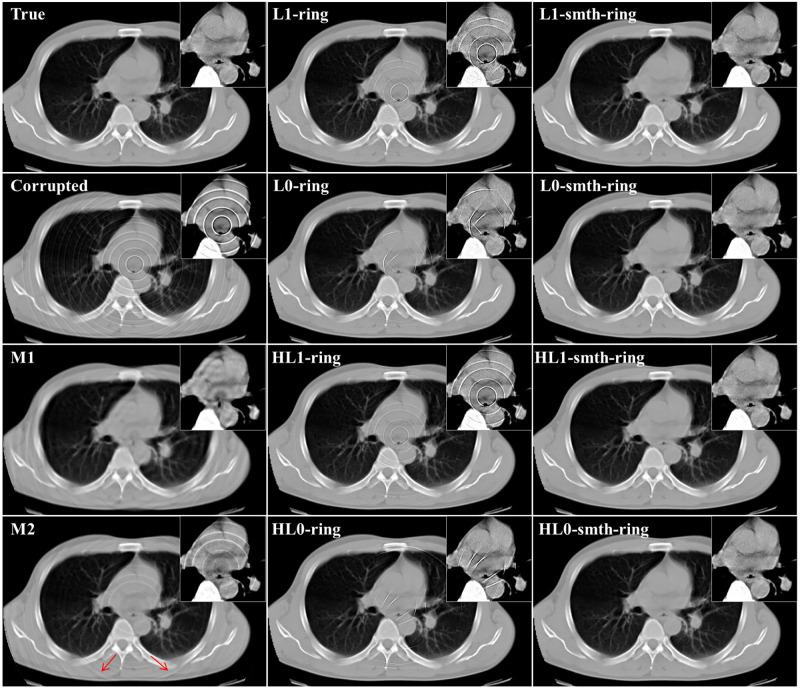
Reconstructed images in the second study case (noise-free, adjacent error detector bins, and angular-independent error case).

**Fig 5:**
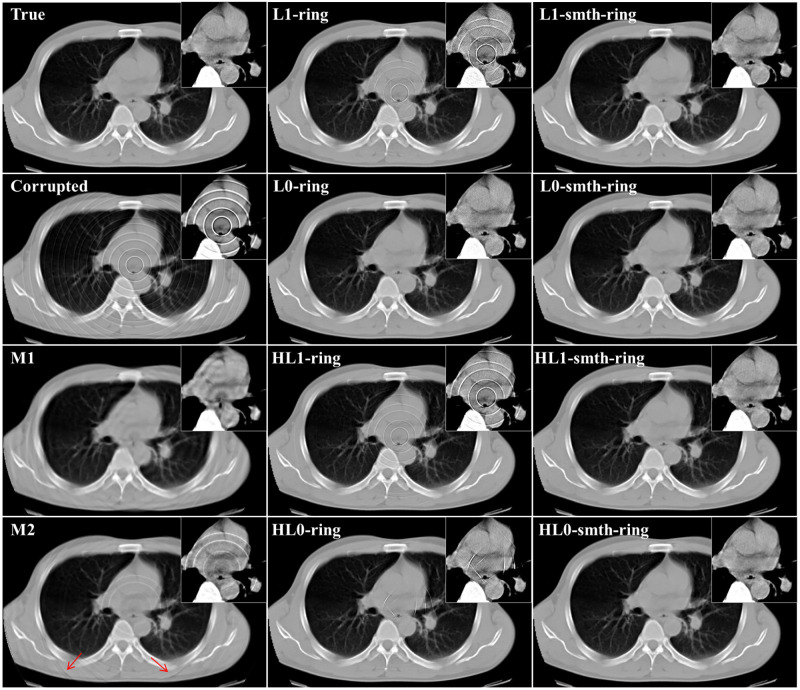
Reconstructed images in the third study case (noise-free, adjacent bad detector bins, and angular-dependent error case).

**Fig 6:**
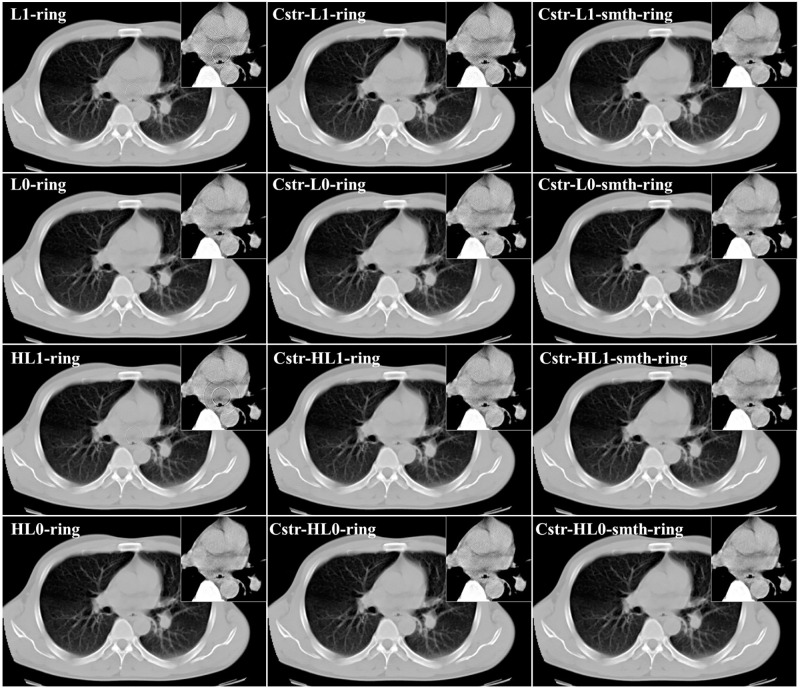
Comparison of reconstruction images between the proposed algorithms and their angular constrained forms for the first study case (noise-free, non-adjacent error detector bins, and angular-independent error case).

**Fig 7:**
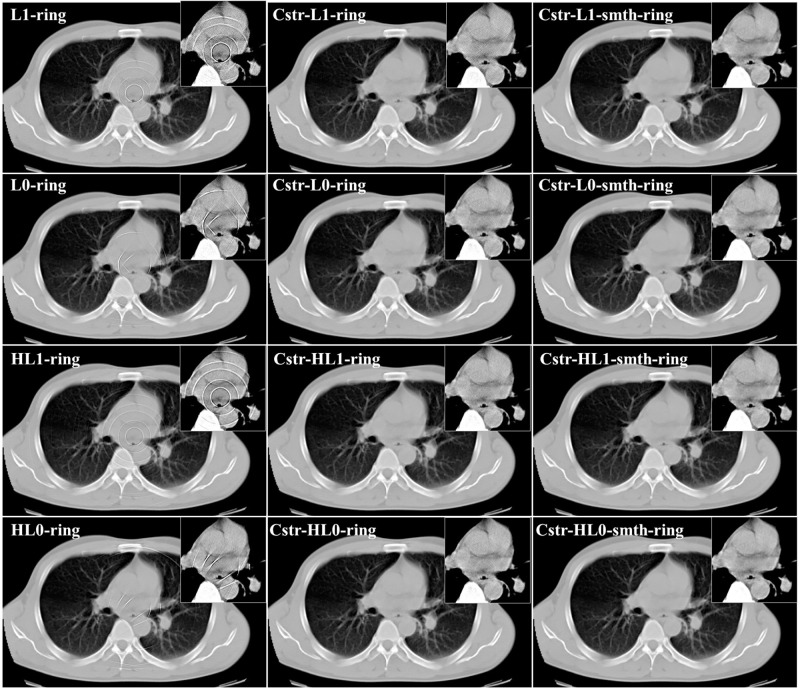
Comparison of reconstructed images between the proposed algorithms and their angular constrained forms for the second study case (noise-free, adjacent error detector bins, and angular-independent error case).

In this section, we applied the proposed algorithms to the simulated projection data without statistical noise corresponding to the above-mentioned 2D chest slice. The simulated sinogram consisted of 512 (bins) × 1,000 (angles), where the standard parallel-beam geometry with 360° angular range was assumed. We considered three different cases, each of which corresponds to a different setup of the non-uniform sensitivity bins and consequently a different ring artifact pattern in the image domain. The details are summarized as follows. In the first case, we assumed that some non-adjacent bins have non-uniform sensitivities producing the ring artifact. In the other two cases, some adjacent detector bins have non-uniform sensitivities producing the thick ring artifact of multiple pixel width where the maximum width was set to 3 (pixels). We assumed that the imperfect error value in each detector bin is constant with respect to the angle in the first and second cases, and varied with respect to the angle in the third case. Using the sinograms $\vec{b}$ corresponding to the three different cases, we reconstructed images $\vec{x}$ by the proposed algorithms as well as M1 and M2 methods. The two parameters (*ρ* and *δ*) control the degree of the ring artifact removal in the proposed algorithms. All the algorithms were implemented using the same values for these parameters *ρ*, *δ*, *N*, and *K* for the following reasons: to obtain a fair comparison between the different algorithms and to show the effect of the different sparsity-induced norms on the ring artifact elimination. The parameter values used in our implementation are summarized as follows: *L* = 2, *K* = 2, 000, *α* = 125 × 10^−3^, *ρ* = 0.004, *δ* = 0.092, $\beta^{\prime }=15.0$, and *η* = 0.001. Also, the parameter values (*σ*=0.8, *L*=4, and *W*=db40) and (*R*=5, *S*_*m*_=5, and *S*_*l*_=10) were used for the correction by M1 and M2 methods, respectively. Fig 2 illustrates an example of the reconstructed images using *ℓ*_2_ and *ℓ*_2_-smth algorithms for the first study case. The true slice, the corrupted image, the reconstructions using all the proposed algorithms, and the images corrected by using the M1 and M2 methods are shown in Figs 3–5 for all the study cases. Moreover, Figs 6 and 7 show the reconstructed images using the angular constrained forms of the proposed algorithms and their improved versions for the first and second study cases, respectively. Also, an enlargement of a small region of interest (ROI) containing the heart region is shown in the upper right corner of each image with more compressed grayscale.

From Fig 2, we can notice that the ring artifact still exists in the reconstructed images using *ℓ*_2_ and *ℓ*_2_-smth algorithms. On the other hand, it is clear from the reconstructions shown in Figs 3–5 that the M1 method eliminated the ring artifact, but there still exist wave-like errors. Meanwhile, the M2 method partially suppressed the ring artifact. Also, additional artifacts were created like the ones shown in the part highlighted by the red arrows. Choosing different parameters in the M1 and M2 methods may further remove the rings but also damages the image so that we did not succeed in getting nicer images than those shown here. By looking at the reconstructions by the proposed algorithms, we can observe that *ℓ*_1_-ring algorithm succeeds in eliminating most of the ring artifact except few rings located near the center. For example, we can notice that one ring appeared with low contrast in the first study case, while very small number of rings still appeared in the second and third study cases. Meanwhile, the reconstructions by *ℓ*_1_-smth-ring algorithm illustrate that the remaining artifacts were smoothed well. The reconstructions by *ℓ*_0_-ring algorithm produced ring-free images, though very little new artifacts were introduced in the second study case. Additional improvement with the smoothing penalty was able to efficiently suppress these remaining artifacts. It also produced high-quality image closer to the original image without any loss in the fine details. Furthermore, the reconstruction by H*ℓ*_1_-ring and H*ℓ*_0_-ring algorithms show that they provide similar results to those in *ℓ*_1_-ring and *ℓ*_0_-ring algorithms, respectively. However, the latter algorithms eliminate more ring artifacts and produce better results than the former ones. The residual artifacts were disappeared well with H*ℓ*_0_-smth-ring algorithm and were smoothed well with H*ℓ*_1_-smth-ring algorithm, where they still slightly appeared with low contrast. Moreover, it is notable from Figs 6 and 7 that the angular constrained forms of the proposed algorithms were able to suppress more ring artifacts compared to the original non-angular constrained ones. It could eliminate approximately all the ring artifact except very few rings appearing with very low contrast in some algorithms. Further improvement with the smoothing penalty can weaken the remaining artifacts.

For quantitative evaluation, we measured the quantitative metrics (RRMSE and SSIM) between the original reference image and the reconstructed image by each algorithm. The obtained metric values are summarized in Tables 2 and 3 for all the study cases. From these results, we can observe that the RRMSE values for the proposed algorithms are significantly smaller than the compared methods. Also, the SSIM values for the proposed algorithms are higher than the compared methods. Furthermore, we note that the metric values for *ℓ*_1_-smth-ring and *ℓ*_0_-smth-ring algorithms and their angular constrained forms outperformed the other proposed algorithms.

**Table 2:** RRMSE and SSIM values of reconstructed images by the proposed algorithms and the compared methods for the first and second study cases and the noisy data.

	Case 1		Case 2		Noisy data	
	RRMSE	SSIM	RRMSE	SSIM	RRMSE	SSIM
M1	0.5852	0.8946	0.5953	0.8926	0.5900	0.8901
M2	0.3184	0.9392	0.3288	0.9304	0.4994	0.9280
*ℓ*_2_	0.4682	0.8757	0.5314	0.8763	0.4695	0.8826
*ℓ*_2_-smth	0.3418	0.9519	0.4741	0.9193	0.3901	0.9429
*ℓ*_1_-ring	0.2968	0.9470	0.3344	0.9374	0.9749	0.6055
*ℓ*_1_-smth-ring	**0.0708**	**0.9969**	**0.0813**	**0.9961**	**0.4342**	**0.9030**
*ℓ*_0_-ring	0.1228	0.9897	0.2215	0.9713	0.8412	0.6974
*ℓ*_0_-smth-ring	**0.0697**	**0.9969**	**0.0757**	**0.9965**	**0.3758**	**0.9429**
H*ℓ*_1_-ring	0.1289	0.9897	0.2396	0.9738	0.8466	0.6943
H*ℓ*_1_-smth-ring	**0.0806**	**0.9961**	**0.0946**	**0.9950**	**0.2928**	**0.9682**
H*ℓ*_0_-ring	0.0837	0.9956	0.2188	0.9749	0.7865	0.7283
H*ℓ*_0_-smth-ring	**0.0754**	**0.9966**	**0.0779**	**0.9963**	**0.3359**	**0.9590**
Cstr-*ℓ*_1_-ring	0.2547	0.9586	0.2474	0.9605	0.9675	0.6165
Cstr-*ℓ*_1_-smth-ring	**0.0705**	**0.9969**	**0.0695**	**0.9969**	**0.5836**	**0.8314**
Cstr-*ℓ*_0_-ring	0.1346	0.9881	0.1309	0.9884	0.9618	0.6190
Cstr-*ℓ*_0_-smth-ring	**0.0746**	**0.9966**	**0.0788**	**0.9963**	**0.5904**	**0.8290**
Cstr-H*ℓ*_1_-ring	0.1272	0.9890	0.1386	0.9870	0.9625	0.6193
Cstr-H*ℓ*_1_-smth-ring	**0.0737**	**0.9966**	**0.0755**	**0.9965**	**0.5853**	**0.8299**
Cstr-H*ℓ*_0_-ring	0.1384	0.9880	0.1324	0.9887	0.9640	0.6183
Cstr-H*ℓ*_0_-smth-ring	**0.0693**	**0.9964**	**0.0775**	**0.9963**	**0.5933**	**0.8270**

**Table 3:** RRMSE and SSIM values of reconstructed images by the proposed algorithms and the compared methods for the third study case.

	Case 3	
	RRMSE	SSIM
M1	0.5957	0.8924
M2	0.3302	0.9294
*ℓ*_2_	0.5370	0.8708
*ℓ*_2_-smth	0.4805	0.9169
*ℓ*_1_-ring	0.3253	0.9403
*ℓ*_1_-smth-ring	**0.0801**	**0.9962**
*ℓ*_0_-ring	0.1445	0.9861
*ℓ*_0_-smth-ring	**0.0751**	**0.9968**
H*ℓ*_1_-ring	0.2403	0.9735
H*ℓ*_1_-smth-ring	**0.0944**	**0.9950**
H*ℓ*_0_-ring	0.1497	0.9871
H*ℓ*_0_-smth-ring	**0.0780**	**0.9963**

**Fig 8:**
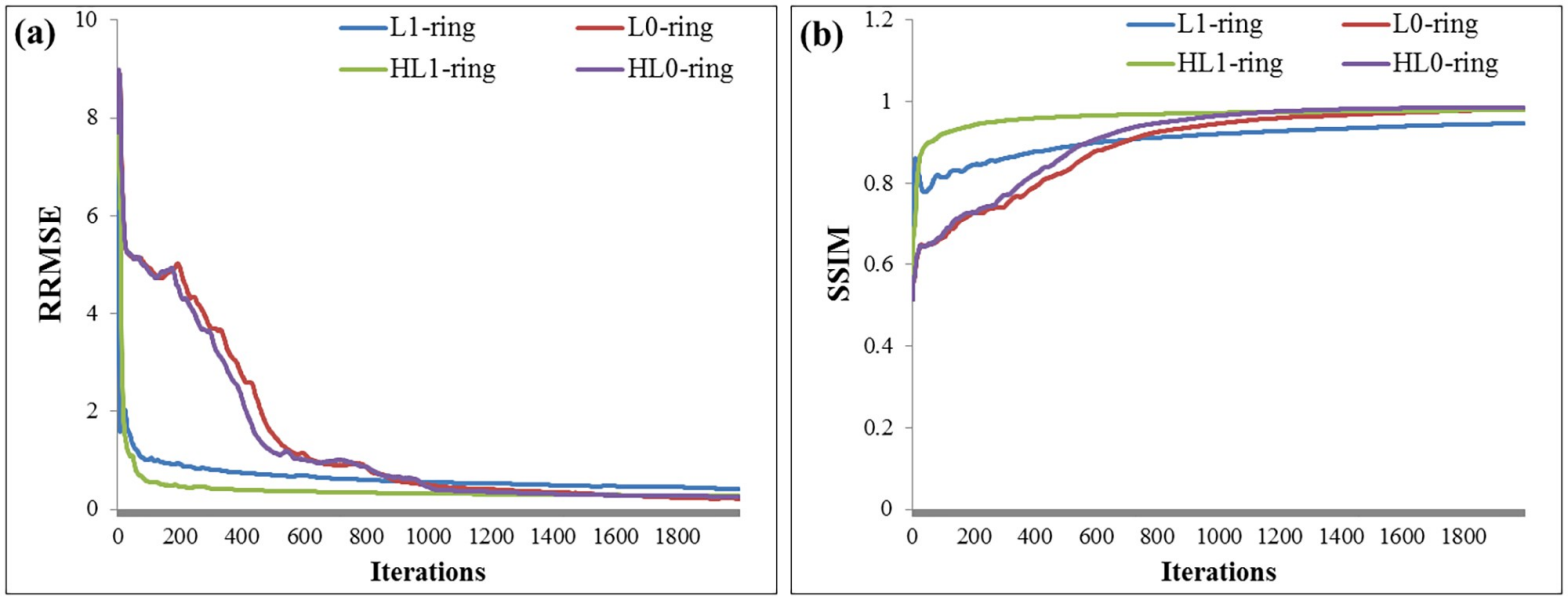
Computed (a) RRMSE and (b) SSIM values versus the iteration numbers for the third study case.

Finally, from the above qualitative and quantitative evaluation, the proposed algorithms and their angular constrained forms, especially with incorporating the smoothing penalty, are effective in suppressing the ring artifact. They also achieve higher image quality compared with the other methods implemented in this work. Fig 8 displays both RRMSE and SSIM values versus the iteration numbers for the third study case, and it is clear that the reconstruction by the proposed algorithms required about 2000 iterations to converge. One drawback of the proposed algorithms is that the iterative algorithms derived from ADMM method are slow in convergence. In the future, we plan to improve the slow convergence speed by introducing faster optimization methods.

### 4.4 Results of the simulation with noisy projection data

**Fig 9:**
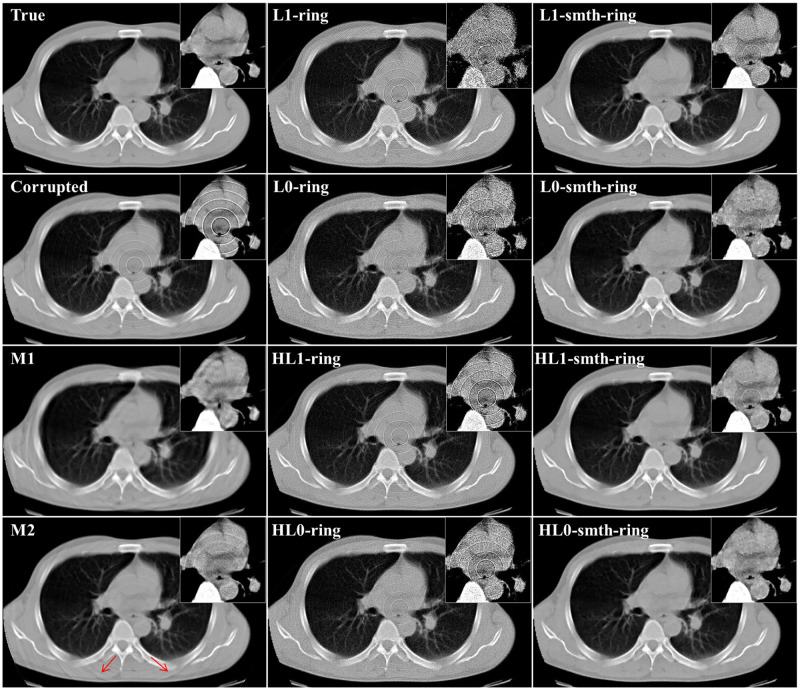
Reconstructed images in the case of noisy projection data (noisy projection data, non-adjacent error detector bins, and angular-independent error case).

**Fig 10:**
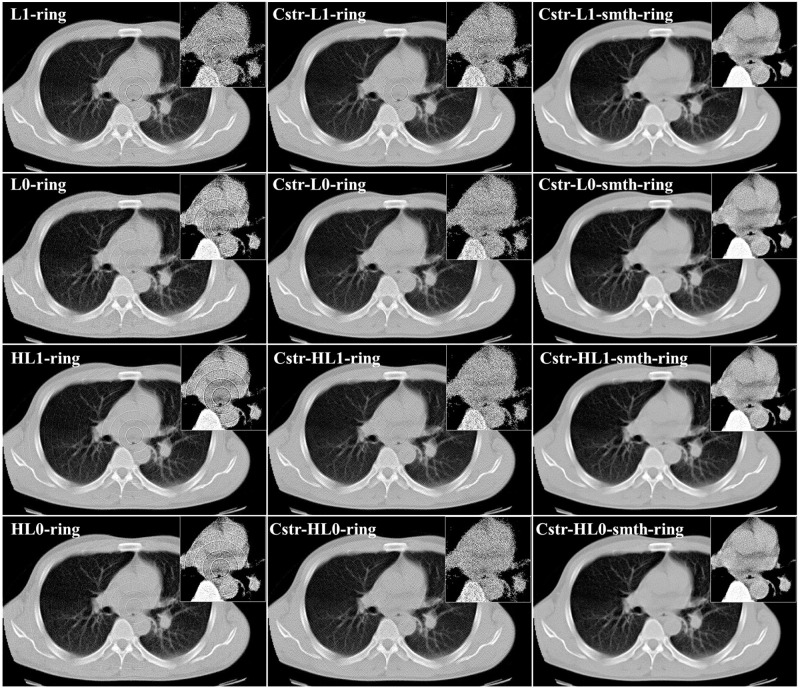
Comparison of reconstructed images between the proposed algorithms and their angular constrained forms in the case of noisy projection data (noisy projection data, non-adjacent error detector bins, and angular-independent error case).

We also studied the case where projection data contains statistical noise in addition to the ring artifact. In the noisy data case, Poisson noise corresponding to 10^6^ (counts/bin) was added to the sinogram of the first study case in the previous section. Using this sinogram, we reconstructed images by the proposed algorithms. The parameter values in the proposed algorithms were as follows: *L* = 2, *K* = 500, *α* = 5 × 10^−3^, *ρ* = 0.001, *δ* = 0.03 and *η* = 0.01. Also, $\beta^{\prime }$ were 750.0 in *ℓ*_1_-smth-ring, 55.0 in *ℓ*_0_-smth-ring, 75.0 in H*ℓ*_1_-smth-ring, 45.0 in H*ℓ*_0_-smth-ring, and 150.0 in the angular constrained forms. Moreover, the parameter values in the M1 and M2 methods were the same as in the case without noise. The true image, the corrupted image, and the reconstructions by the proposed algorithms and the compared methods are shown in Figs 9 and 10 shows the reconstructions by the angular constrained forms of the proposed algorithms and their improved versions with the smoothing penalty.

From the visual evaluation of the results, we can notice that the M1 and M2 methods produced similar results to those in the previously studied noise-free case. Furthermore, we can observe that the reconstructions by *ℓ*_1_-ring and *ℓ*_0_-ring algorithms did not succeed in completely eliminating the ring artifact, where it only slightly decreased the intensity of some rings. The reconstructions by H*ℓ*_1_-ring and H*ℓ*_0_-ring algorithms produced similar results to those in *ℓ*_1_-ring and *ℓ*_0_-ring algorithms, respectively. Meanwhile, the remaining artifacts were weakened with *ℓ*_1_-smth-ring algorithm. Also, they were suppressed with *ℓ*_0_-smth-ring, H*ℓ*_1_-smth-ring, and H*ℓ*_0_-smth-ring, where the artifact slightly appeared with low contrast. It can be notable that the reconstructions by H*ℓ*_1_-ring and H*ℓ*_0_-ring algorithms contained less noise compared to *ℓ*_1_-ring and *ℓ*_0_-rings algorithms. The reason for this is the existence of the quadratic part in Huber *ℓ*_1_-norm and Huber *ℓ*_0_-norm cost functions in H*ℓ*_1_-ring and H*ℓ*_0_-ring algorithms.

Concerning the results of the angular constrained forms of the proposed algorithms, it is clear that the angular constrained forms of the proposed algorithm could eliminate all the ring artifact with *ℓ*_0_-ring and H*ℓ*_0_-ring algorithms. They also removed most of the rings except some rings located near the center in both *ℓ*_1_-ring and H*ℓ*_1_-ring algorithms, although some new artifacts were introduced. Additionally, we can observe that the angular constrained forms of the improved algorithms could effectively remove the artifact and produce ring-free images.

To perform quantitative evaluation of the proposed algorithms, RRMSE and SSIM values were measured and presented in the last two columns in Table 2. We can observe that the improved versions of the proposed algorithms and their angular constrained forms achieved the best values among all the implemented algorithms. The performance of the proposed algorithms in the case of noisy projection is less than that in the case of noise-free projection data, even with H*ℓ*_1_-ring or H*ℓ*_0_-ring algorithms. Consequently, we plan to incorporate a statistical model in addition to the sparsity-induced norms for further improvement of the reconstruction with noisy projection data.

### 4.5 Results of the real data

**Fig 11:**
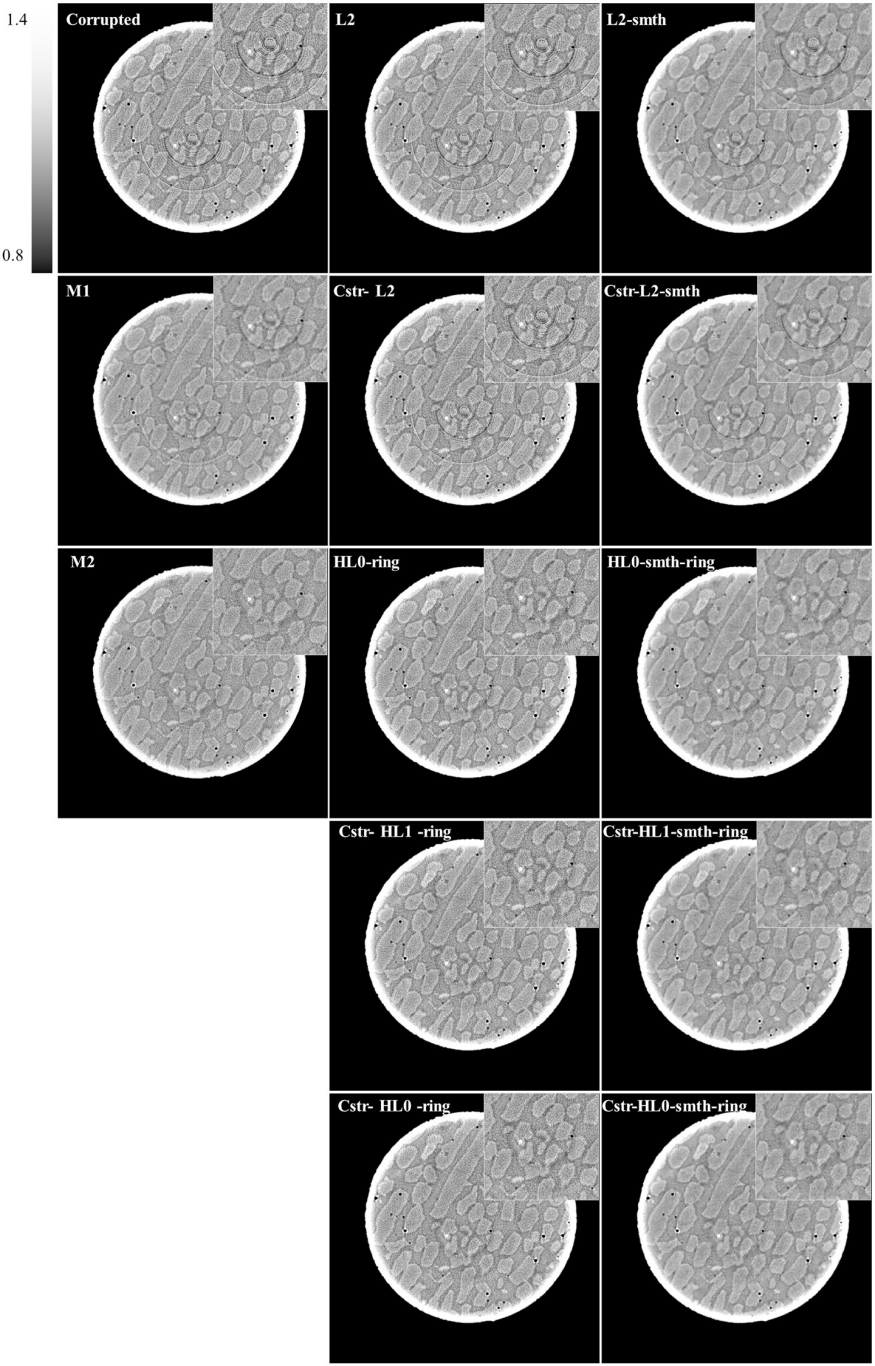
Results of the real data.

For additional evaluation of the proposed algorithms, we implemented the proposed algorithms with real data. We used the projection data corresponding to the central slice of the above-mentioned real data. We implemented the proposed method and the compared methods with this data. The results are shown in Fig 11. Also, an enlargement of a small ROI located in the center of each reconstruction is shown in the upper right corner of each image.

The results are summarized as follows. Most of the ring artifacts were removed well with the corrections using M1 and M2 methods. However, some remaining rings were still visible. These existing techniques worked well, but were not complete in this experiment. The reconstructed images with *ℓ*_2_ and *ℓ*_2_-smth algorithms and their angular constrained versions (cstr-*ℓ*_2_ and cstr-*ℓ*_2_-smth) still included the strong ring artifact. Since the real projection data is corrupted with noise, H*ℓ*_0_-ring, H*ℓ*_0_-smth-ring, and the angular constraint versions of H*ℓ*_1_-ring and H*ℓ*_0_-ring algorithms could remove most of the ring artifact well. Moreover, cstr-smth-H*ℓ*_1_-ring and cstr-smth-H*ℓ*_0_-ring algorithms almost completely removed the ring artifact leading to high-quality reconstructions. Finally, we would like to strengthen the following fact. Using *ℓ*_2_-norm cannot suppress the ring artifact, whereas the combination of *ℓ*_1_-or *ℓ*_0_-norm and *ℓ*_2_-norm as in Huber *ℓ*_1_- and Huber *ℓ*_0_-norms is able to remove the ring artifact. This means that the use of sparsity-induced norm is essential in the ring-artifact elimination problem.

## 5 Conclusion

In this work, we developed a class of algorithms to eliminate the ring artifact from CT images. The first group of algorithms minimizes different sparsity-induced norms of the imperfect error components of the sinogram. In the second group of algorithms, we improved the first class of algorithms by incorporating the smoothing penalty into the cost function to remove the remaining ring artifact. Furthermore, we also introduced angular constrained forms corresponding to the proposed algorithms and their improved versions. The proposed algorithms are able to correct the ring artifact well during image reconstruction and need neither pre-processing nor post-processing steps. We evaluated the performance of these algorithms through a number of simulation and real data studies. In the simulation, we considered three different cases where different setups of the ring artifact patterns were simulated. Both the qualitative and quantitative evaluation results demonstrated that the improved versions of the proposed algorithms and their angular constrained forms are efficient in ring artifact suppression. They also provide significantly better results than the compared methods, *i.e.* M1 and M2 methods. In summary, we can conclude that the sparsity-induced norms for the imperfect error components of the projection data in the data fidelity as well as the smoothing penalty can effectively eliminate the ring artifact.

## Supporting information

S1 File (RAR)

## References

[1] Boas FE, Fleischmann D. CT artifacts: causes and reduction techniques. Imaging in medicine. 2012;4(2):229–240.

[2] Sijbers J, Postnov A. Reduction of ring artefacts in high resolution micro-CT reconstructions. Physics in Medicine Biology. 2004;49(14):N247. doi: 10.1088/0031-9155/49/14/N06 15357205

[3] Cand‘es EJ, Wakin MB. An introduction to compressive sampling. IEEE signal processing magazine. 2008;25(2):21–30. doi: 10.1109/MSP.2007.914731

[4] Yan L, Wu T, Zhong S, Zhang Q. A variation-based ring artifact correction method with sparse constraint for flat-detector CT. Physics in Medicine Biology. 2016;61(3):1278. doi: 10.1088/0031-9155/61/3/1278 26789081

[5] Li Y, Zhao Y, Ji D, Lv W, Xin X, Zhao X, et al. Sparse-domain regularized stripe decomposition combined with guided-image filtering for ring artifact removal in propagation-based x-ray phase-contrast CT. Physics in Medicine Biology. 2021;66(10):105011. doi: 10.1088/1361-6560/abf9de 33878737

[6] Chao Z, Kim HJ. Removal of computed tomography ring artifacts via radial basis function artificial neural networks. Physics in Medicine Biology. 2019;64(23):235015. doi: 10.1088/1361-6560/ab5035 31639777

[7] Paleo P, Mirone A. Ring artifacts correction in compressed sensing tomographic reconstruction. Journal of synchrotron radiation. 2015;22(5):1268–1278. doi: 10.1107/S1600577515010176 26289279 PMC4787841

[8] Salehjahromi M, Wang Q, Zhang Y, Gjesteby LA, Harrison D, Wang G, et al. A new iterative algorithm for ring artifact reduction in CT using ring total variation. Medical physics. 2019;46(11):4803–4815. doi: 10.1002/mp.13762 31408539 PMC7393670

[9] Kudo H, Takaki K, Yamazaki F, Nemoto T. Proposal of fault-tolerant tomographic image reconstruction. In: Developments in X-Ray Tomography X. vol. 9967. International Society for Optics and Photonics; 2016. p. 99671K.

[10] Kudo H, Dong J, Chigita K, Kim Y. Metal artifact reduction in CT using fault-tolerant image reconstruction. In: Developments in X-Ray Tomography XII. vol. 11113. International Society for Optics and Photonics; 2019. p. 111130A.

[11] Chigita K, Dong J, Kudo H. An iterative reconstruction method for CT metal artifact reduction using L1 norm data fidelity and nonlocal TV regularization. In: International Forum on Medical Imaging in Asia 2021. vol. 11792. International Society for Optics and Photonics; 2021. p. 117920Z.

[12] Pelt DM, Parkinson DY. Ring artifact reduction in synchrotron x-ray tomography through helical acquisition. Measurement Science and Technology. 2018;29(3):034002. doi: 10.1088/1361-6501/aa9dd9

[13] Jenneson PM, Gilboy WB, Morton EJ, Gregory PJ. An X-ray micro-tomography system optimised for the low-dose study of living organisms. Applied radiation and isotopes. 2003;58(2):177–181. doi: 10.1016/S0969-8043(02)00310-X 12573316

[14] Münch B, Trtik P, Marone F, Stampanoni M. Stripe and ring artifact removal with combined wavelet—Fourier filtering. Optics express. 2009;17(10):8567–8591. doi: 10.1364/OE.17.008567 19434191

[15] Titarenko S, Titarenko V, Kyrieleis A, Withers PJ. A priori information in a regularized sinogram-based method for removing ring artefacts in tomography. Journal of synchrotron radiation. 2010;17(4):540–549. doi: 10.1107/S0909049510010964 20567087

[16] Anas EMA, Lee SY, Hasan MK. Removal of ring artifacts in CT imaging through detection and correction of stripes in the sinogram. Physics in Medicine Biology. 2010;55(22):6911. doi: 10.1088/0031-9155/55/22/020 21048294

[17] Hasan MK, Sadi F, Lee SY. Removal of ring artifacts in micro-CT imaging using iterative morphological filters. Signal, Image and Video Processing. 2012;6(1):41–53. doi: 10.1007/s11760-010-0170-z

[18] Kim Y, Baek J, Hwang D. Ring artifact correction using detector line-ratios in computed tomography. Optics express. 2014;22(11):13380–13392. doi: 10.1364/OE.22.013380 24921532

[19] Vo NT, Atwood RC, Drakopoulos M. Superior techniques for eliminating ring artifacts in X-ray micro-tomography. Optics express. 2018;26(22):28396–28412. doi: 10.1364/OE.26.028396 30470012

[20] Titarenko V. 1-D filter for ring artifact suppression. IEEE Signal Processing Letters. 2016;23(6):800–804. doi: 10.1109/LSP.2016.2554363

[21] Guo H, Zeng D, Zhang H, Huang J, Zhang J, Ma J. CT ring artifact reduction using an improved wavelet filtering in the sinogram domain. Journal of Southern Medical University. 2015;35(9):1258–1262. 26403734

[22] Ashrafuzzaman A, Lee SY, Hasan MK. A self-adaptive approach for the detection and correction of stripes in the sinogram: suppression of ring artifacts in CT imaging. EURASIP Journal on Advances in Signal Processing. 2011;2011:1–13. doi: 10.1155/2011/183547

[23] Nauwynck M, Bazrafkan S, Van Heteren A, De Beenhouwer J, Sijbers J, München ZS, et al. Ring artifact reduction in sinogram space using deep learning. In: The International Conference on Image Formation in X-ray Computed Tomography. Regensburg, Germany 2020; 2020.

[24] Prell D, Kyriakou Y, Kalender WA. Comparison of ring artifact correction methods for flat-detector CT. Physics in Medicine Biology. 2009;54(12):3881. doi: 10.1088/0031-9155/54/12/018 19491452

[25] Zhao S, Li J, Huo Q. Removing ring artifacts in CBCT images via generative adversarial network. In: 2018 IEEE International Conference on Acoustics, Speech and Signal Processing (ICASSP). IEEE; 2018. p. 1055–1059.

[26] Liang X, Zhang Z, Niu T, Yu S, Wu S, Li Z, et al. Iterative image-domain ring artifact removal in cone-beam CT. Physics in Medicine Biology. 2017;62(13):5276. doi: 10.1088/1361-6560/aa7017 28585520

[27] Chen YW, Duan G, Fujita A, Hirooka K, Ueno Y. Ring artifacts reduction in cone-beam CT images based on independent component analysis. In: 2009 IEEE Instrumentation and Measurement Technology Conference. IEEE; 2009. p. 1734–1737.

[28] Wei Z, Wiebe S, Chapman D. Ring artifacts removal from synchrotron CT image slices. Journal of Instrumentation. 2013;8(06):C06006. doi: 10.1088/1748-0221/8/06/C06006

[29] Wu H, Li J, Wang H. Removing ring artifacts in cone-beam CT via TV-Stokes and unidirectional total variation model. Medical physics. 2019;46(4):1719–1727. doi: 10.1002/mp.13430 30723939

[30] Huo Q, Li J, Lu Y. Removing ring artefacts in CT images via unidirectional relative variation model. Electronics Letters. 2016;52(22):1838–1839. doi: 10.1049/el.2016.2692

[31] Wang Z, Li J, Enoh M. Removing ring artifacts in CBCT images via generative adversarial networks with unidirectional relative total variation loss. Neural Computing and Applications. 2019;31(9):5147–5158. doi: 10.1007/s00521-018-04007-6

[32] Boyd S, Parikh N, Chu E, Peleato B, Eckstein J, et al. Distributed optimization and statistical learning via the alternating direction method of multipliers. Foundations and Trends® in Machine learning. 2011;3(1):1–122. doi: 10.1561/2200000016

[33] Rashed EA, al Shatouri M, Kudo H. Sparsity-constrained three-dimensional image reconstruction for C-arm angiography. Computers in biology and medicine. 2015;62:141–153. doi: 10.1016/j.compbiomed.2015.04.014 25932971

[34] Thibault JB, Sauer KD, Bouman CA, Hsieh J. A three-dimensional statistical approach to improved image quality for multislice helical CT. Medical physics. 2007;34(11):4526–4544. doi: 10.1118/1.2789499 18072519

[35] Toda H. X-ray CT: Practical use of tomography in industry and science and engineering. Kyoritsu Shuppan; 2019 (in Japanese).

[36] Toda H, Tomizato F, Harasaki R, Seo D, Kobayashi M, Takeuchi A, et al. 3D fracture behaviours in dual-phase stainless steel. ISIJ international. 2016;56(5):883–892. doi: 10.2355/isijinternational.ISIJINT-2015-631

[37] Hsieh J. Computed tomography: principles, design, artifacts, and recent advances. vol. 114. SPIE press; 2003.

[38] Wang Z, Bovik AC, Sheikh HR, Simoncelli EP. Image quality assessment: from error visibility to structural similarity. IEEE transactions on image processing. 2004;13(4):600–612. doi: 10.1109/TIP.2003.819861 15376593

